# Integrating Line Transect Distance Sampling and Spatial Analysis to Assess Local Density and Habitat Use of *Capra aegagrus* in Batman Province, Türkiye

**DOI:** 10.3390/life16030432

**Published:** 2026-03-06

**Authors:** Eyüp Yıldırım, Servet Ulutürk

**Affiliations:** 1 Institute of Graduate Studies, University of Batman, 72070 Batman, Türkiye; eyyup-76@hotmail.com; 2 Department of Biology, Faculty of Science and Arts, Batman University, 72070 Batman, Türkiye

**Keywords:** biodiversity, distance sampling, habitat use, local density, wild goats, spatial ecology, Türkiye

## Abstract

Understanding local population density and spatial habitat use is essential for wildlife conservation in fragmented mountainous landscapes. This study examined the habitat use patterns of *Capra aegagrus* in the mountainous regions of Batman, Türkiye, using Kernel Density Estimation (KDE) and spatial regression modeling. Significant spatial autocorrelation (Moran’s I = 0.799, *p* < 0.001) justified the use of a Spatial Error Model (AIC = −254.59). Built land proportion had a strong negative effect, with a 10% increase associated with a 31% decline in KDE intensity. Elevation also showed a modest negative association with habitat use intensity, whereas slope and bare land proportion were positively associated. The southern stratum exhibited higher relative encounter intensity, and the spatial autoregressive parameter (λ = 0.92) indicated strong spatial structuring. To complement spatial habitat analysis with demographic estimates, population density was assessed using Line Transect Distance Sampling in the northern and southern sub-regions. The estimated local density was 6.47 individuals/km^2^ (95% CI: 4.11–10.16), with overlapping confidence intervals between sub-regions. The variation in detection probability and encounter rate contributed the most to overall uncertainty. Because the surveys were restricted to accessible mountainous terrain, estimates represent local ecological density rather than province-wide abundance. Together, these results provide a spatially explicit baseline linking relative habitat use patterns with locally derived density estimates to support future monitoring and conservation planning.

## 1. Introduction

The wild goat (*Capra aegagrus*) is classified as Near Threatened by the International Union for Conservation of Nature (IUCN) and inhabits mountainous regions across the Middle East and the Caucasus, particularly within highland systems surrounding the Mediterranean Basin [1,2]. In Türkiye, wild goats occupy rugged terrains extending from the Datça Peninsula in the west through the Taurus and Anti-Taurus mountain systems to eastern and south-eastern Anatolia, occupying elevations from sea level up to approximately 3000 m [3,4,5]. Previous studies have demonstrated that altitude, slope, terrain ruggedness, and vegetation structure strongly influence wild goats’ distribution and habitat selection [6,7].

Beyond its intrinsic conservation value, *C. aegagrus* plays an important ecological role as a key ungulate in semi-arid mountain ecosystems and it serves as prey for large carnivores, including the Anatolian leopard and the gray wolf. Fluctuations in these goats’ abundance may therefore influence predator dynamics and broader trophic interactions [7,8,9,10].

Wildlife is crucial to ecosystems and helps us to understand species’ geographic ranges, which are vital for conserving biodiversity [11]. To achieve effective wildlife conservation, reliable information on population abundance and spatial distribution is required, particularly for large mammals inhabiting rugged and fragmented landscapes [12,13,14,15]. Estimating the abundance of mountain ungulates presents logistical and methodological challenges due to steep terrain, heterogeneous visibility, and limited accessibility. Among available approaches, Line Transect Distance Sampling (LTDS) has become widely applied for density estimation, as it accounts for imperfect detection and provides statistically robust estimates when key assumptions are met [16,17].

In addition to line transect approaches, Camera-Trap Distance Sampling (CTDS) has been developed as an alternative framework for density estimation, particularly where camera-based detection is preferred [18,19,20,21,22,23,24,25,26,27]. CTDS can enable multi-species monitoring and reduce observer-related bias; however, its application depends on specific assumptions regarding camera placement and detection geometry. Each method presents distinct advantages and limitations depending on terrain structure, detectability patterns, and logistical feasibility. The present study focuses exclusively on LTDS within the surveyed mountainous habitats.

In recent years, new techniques have been developed to complement traditional field research methods in wildlife monitoring, including automated detection and artificial intelligence-based approaches such as deep learning object detection models, computer vision systems, and electronic tracking technologies. These enable high-accuracy detection of wild animals from camera-trap images, as well as visual analyses and electronic monitoring, providing real-time movement and behaviour data for ecological studies [28,29,30].

In addition to density estimation, spatial analytical tools such as Kernel Density Estimation (KDE) can be used to describe relative encounter intensity and spatial use patterns within surveyed areas. Distance-based metrics may further help to explore the associations between observed encounters and selected environmental features. Although numerous studies in Türkiye have documented the distribution and habitat preferences of *C. aegagrus*, region-specific density estimates remain limited, particularly in south-eastern Anatolia. Given that there are increased habitat fragmentation and anthropogenic pressures in mountainous landscapes, locally grounded assessments are needed to inform regional conservation planning.

Although numerous studies in Türkiye have examined the morphological traits, distribution ranges, and habitat preferences of wild goats, region-specific quantitative density estimates have increased in recent years [31,32,33,34,35,36,37,38,39,40,41,42]. This region represents a biogeographically transitional and increasingly fragmented mountainous landscape, where locally grounded assessments are needed to inform conservation planning and long-term monitoring strategies. The primary objective of this study was to estimate the local population density of *C. aegagrus* within surveyed mountainous habitats in Batman Province using Line Transect Distance Sampling. In addition, we aimed to characterize spatial patterns of habitat use by integrating Kernel Density Estimation and distance-based environmental analyses. Rather than producing a province-wide predictive habitat suitability model, this study focuses on describing ecological density and habitat use patterns within accessible mountainous areas where systematic surveys were conducted.

## 2. Materials and Methods

### 2.1. Study Area

The study was conducted in Batman Province, South-Eastern Türkiye, which covers approximately 4349 km^2^. However, the field surveys were restricted to accessible mountainous habitats, bringing the total to 431 km^2^ (190 km^2^ in the northern stratum and 241 km^2^ in the southern stratum). The province is characterized by mountainous terrain along its northern and southern boundaries, separated by the Tigris River basin, which comprises fertile plains, settlements, and agricultural land. The northern section includes the Mereto, Sultan, and Aydınlık mountains, with Mereto Mountain reaching 2967 m, while the southern boundary is defined by the Raman and Karakaş mountain systems [43].

**Figure 1:**
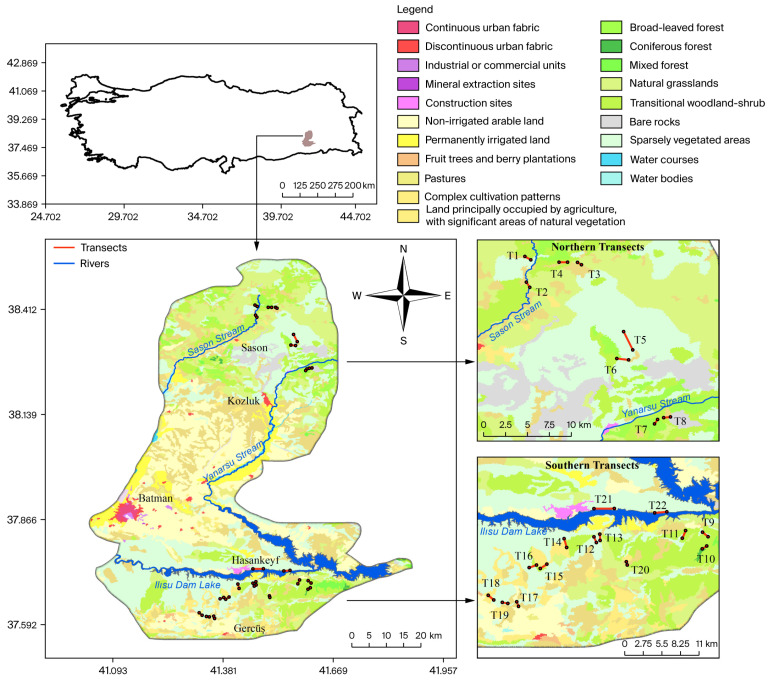
Location of the study area and spatial distribution of line transects used for wild goat (*Capra aegagrus*) surveys in Batman Province, South-Eastern Türkiye. The map shows the position of Batman Province within Türkiye, land cover classes derived from CORINE data, major rivers and the Ilısu Dam reservoir, and the division of line transects into northern and southern survey sections. Transect lines and observation points indicate areas where LTDS was conducted to assess population distribution and habitat use.

The region exhibits a continental climate and is predominantly covered by steppe vegetation with Irano-Turanian floristic elements. A major hydrogeological feature is the Ilısu Dam reservoir, operational since 2020, which bisects the landscape and potentially influences wildlife movement and connectivity. To account for this landscape fragmentation, the study area was stratified into northern and southern mountainous sub-regions (Figure 1).

### 2.2. Transect Design and Sampling Strategy

Field surveys were conducted between October 2020 and May 2023. A total of 22 line transects were established: 8 in the northern region and 14 in the southern region. Transect lengths ranged from 469 m to 2625 m (Table S1). Due to complex topography, logistical constraints, and security restrictions, fully randomized transect placement across the entire province was not feasible. Therefore, a constrained systematic sampling approach was implemented within accessible mountainous habitats known to support *Capra aegagrus*. Low-lying plains and highly urbanized areas were intentionally excluded because they are not suitable habitats for this montane species. Also, certain areas were inaccessible due to military restrictions. Consequently, the density estimates derived from this design represent local ecological density within surveyed mountainous habitats rather than province-wide abundance. Each transect was surveyed 11 times, resulting in 242 total walks and a cumulative sampling effort of 283.47 km. However, such adaptive sampling strategies are widely accepted in wildlife studies conducted in rugged terrain where strict randomization is practically impossible [44,45].

### 2.3. Field Data Collection

Surveys were conducted between sunrise and sunset by two trained observers. For each detection, radial distance and sighting angle were measured using a laser rangefinder, and perpendicular distances were calculated using trigonometric conversion. Group size and demographic composition were recorded. A total of 87 independent encounters were recorded (30 in the northern region and 57 in the southern region). This sample size meets the commonly recommended thresholds for distance sampling analyses, as it exceeds the minimum of 60–80 encounters reported in the literature [46,47,48]. Following truncation procedures applied during detection modelling, 65 encounters (22 North, 43 South) were retained for density estimation.

### 2.4. Field Population Density Estimation

Population density was estimated using Line Transect Distance Sampling (LTDS) in Rv.4.5.2 (R Foundation for Statistical Computing, Vienna, Austria), implemented with the Distance package. Perpendicular distances were modelled to estimate detection probability. Detection distances (r) and sighting angles (Ɵ) recorded during field surveys were converted into perpendicular distances (x) using the trigonometric formula (x = r sin Ɵ) [16]. To improve model fit and reduce potential bias from movement near the transect line, left truncation at 50 m and right truncation of the furthest 10% of observations were applied. Candidate detection functions were evaluated, and the half-normal key function with cosine adjustment terms was selected based on Akaike’s Information Criterion (AIC) and goodness-of-fit diagnostics (Cramér–von Mises test). Given the constrained transect design, the analyses focused on estimating local density within the surveyed mountainous habitats rather than trying to extrapolate them to the entire province. The theoretical and mathematical background of the LTDS method has been described in detail previously [44].

### 2.5. Spatial Habitat Use Analysis

Spatial patterns of habitat use were characterized using Kernel Density Estimation (KDE) derived from encounter locations. KDE quantified relative encounter intensity and visualized spatial variation within surveyed mountainous areas. Initial bandwidth selection followed rule-of-thumb estimators based on the spatial dispersion (standard deviation) of encounter coordinates, yielding values of 3.2 km (north) and 3.4 km (south). However, the preliminary outputs produced highly fragmented, locally over-fitted density surfaces. To better represent landscape-scale patterns and reduce sensitivity to individual observations, a 12 km bandwidth was selected. This bandwidth approximates the spatial extent of mountain systems and reflects landscape-level habitat structure rather than fine-scale clustering of individual sightings. Sensitivity analyses using alternative bandwidths (3 km and 8 km) showed consistent overall spatial patterns, supporting the robustness of the chosen bandwidth.

To enable direct comparison between the northern and southern strata, a common intensity threshold (the mean + SD of the higher-density stratum) was applied to both KDE surfaces. KDE values were log-transformed prior to modelling to improve normality and meet linear regression assumptions. The environmental predictors included elevation (m), slope (degrees), land-cover proportions (water, built, trees, crop, and bare areas), and a categorical stratum variable (North vs. South). Multicollinearity was assessed using Variance Inflation Factors (VIFs), and all predictors were retained because VIFs were within acceptable limits.

Competing models (OLS, SAR, SDM, and SEM) were compared using AIC. The reduced SEM, obtained by removing non-significant predictors, showed the lowest AIC (−254.59); therefore, it was selected as the final model. Spatial autocorrelation in model residuals was assessed using Moran’s I. Because the dependent variable was log-transformed, regression coefficients were back-transformed using exp(β) − 1 to express effects as proportional percentage changes in habitat use intensity. All spatial analyses were conducted in R using the terra and geodata packages. The complete analytical workflow is archived on Zenodo https://doi.org/10.5281/zenodo.18374659 (accessed on 6 November 2025).

## 3. Results

Between October 2020 and May 2023, local population density and spatial habitat use patterns of *Capra aegagrus* were investigated using Line Transect Distance Sampling (LTDS) and Kernel Density Estimation (KDE). The survey efforts covered 283.47 km of transects within a 431 km^2^ mountainous study area, with an effectively sampled area of 112.99 km^2^. A total of 806 individuals were recorded across 87 initial encounters, including 461 juveniles, 73 males, and 272 females (Table 1 and Table S1).

### 3.1. Population Density Estimation

After applying left truncation (50 m) and right truncation (10%), 65 encounters were retained for detection modelling (22 in the north and 43 in the south; Table 1). The half-normal key function with cosine adjustment provided the most parsimonious fit, as indicated by AIC and goodness-of-fit diagnostics. The estimated average detection probability (*p* = 0.78, SE = 0.140, CV = 18.07%) indicates satisfactory detectability within the surveyed mountainous habitats. The effective strip width (ESW) was estimated at 155.31 m (Table 2).

The overall encounter rate of clusters [ER(S)] was 0.23 per km (CV = 11.37%), indicating relatively consistent group detection across the transects. The density of individuals for the total study area was estimated at: D = 6.47 individuals/km^2^ (SE = 1.498, CV = 23.17%, 95% CI: 4.11–10.16). The corresponding local population size within the surveyed area was N = 2787 individuals (SE = 645.79, CV = 23.17%). At the regional scale, this was north: D = 5.85 individuals/km^2^ (CV = 39.82%) and south: D = 6.95 individuals/km^2^ (CV = 28.06%) (Table 2). Density-related parameters exhibited higher coefficients of variation in the northern stratum, reflecting reduced precision associated with a smaller post-truncation sample size (*n* = 22).

**Figure 2:**
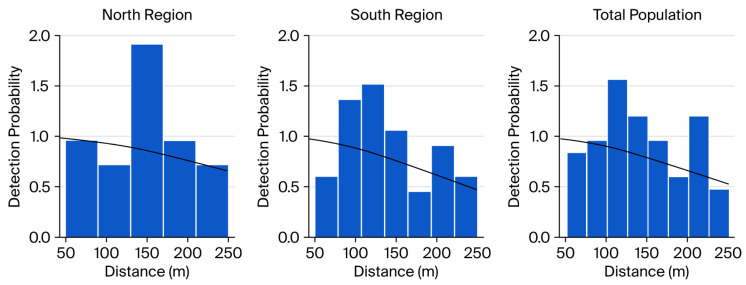
Distance histograms and fitted detection functions obtained from the Line Transect Distance Sampling (LTDS) analysis for *Capra aegagrus*. Panels show results for the north, south, and total study areas. Bars represent the frequency of observations by distance class after truncation, while solid lines indicate the fitted detection functions, illustrating the decline in detection probability with increasing distance from the transect line.

Distance histograms and fitted detection functions (Figure 2) indicated adequate model fit in both north and south strata after truncation, with detection probability declining smoothly with increasing perpendicular distance and no evident deviation from distance sampling assumptions.

### 3.2. Spatial Patterns of Habitat Use

**Figure 3:**
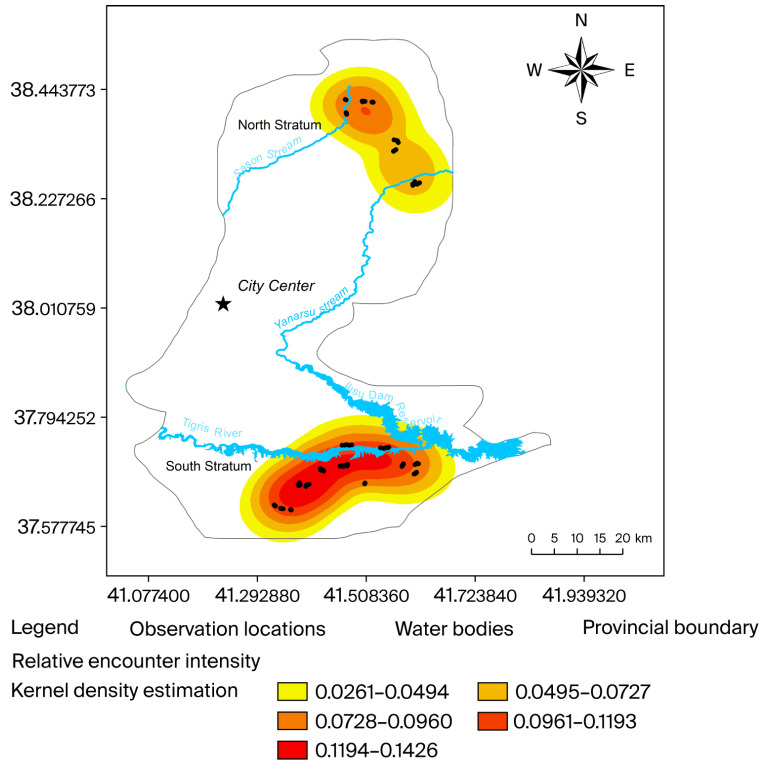
Kernel Density Estimation (KDE) map showing relative encounter intensity of *Capra aegagrus* in the northern and southern mountainous strata of Batman Province, Türkiye. Warmer colours indicate higher encounter intensity, and black points represent observation locations from line transect surveys. The Ilısu Dam reservoir separates the two strata. KDE values reflect relative use within surveyed areas only.

Kernel Density Estimation (KDE) was used to characterize relative spatial encounter intensity across surveyed mountainous habitats. KDE values ranged from 0.056 to 0.143 (mean = 0.101). Spatial surfaces revealed localized hotspots of encounter concentration in both northern and southern mountain systems (Figure 3). Areas outside the surveyed mountainous regions showed low KDE values, reflecting limited sampling rather than confirmed ecological absence.

The OLS model showed strong and significant spatial autocorrelation in residuals (Moran’s I = 0.72, *p* < 0.001; Table S2), justifying the use of spatial regression models. All the spatial models substantially improved model fit compared to OLS, as indicated by markedly lower AIC values (Table S2). Among them, the reduced Spatial Error Model (SEM) showed the lowest AIC (−254.59); therefore, it was selected as the final model. The spatial error parameter was high and highly significant (λ = 0.92, *p* < 0.001; Table 3), indicating strong spatial structure in the error term. Although spatial autocorrelation was reduced relative to OLS, a weak residual spatial structure remained. Descriptive statistics of KDE intensity and environmental predictors are presented in Supplementary Table S3.

**Figure 4:**
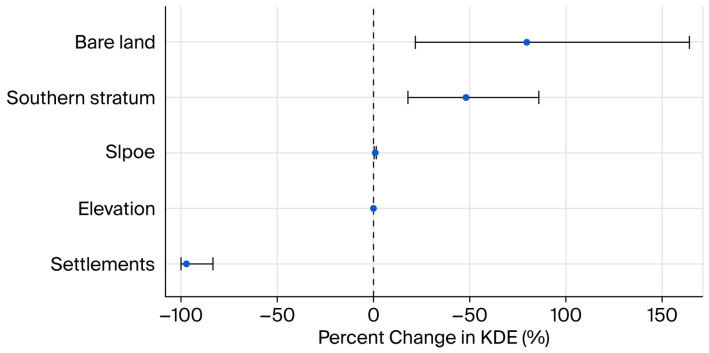
Percentage change in KDE intensity associated with predictors retained in the final reduced Spatial Error Model. Effects are expressed as percent change in KDE for a 100 m increase in elevation, 1° increase in slope, and 10% increase in land-cover variables. Error bars represent 95% confidence intervals.

In the final model (Table S2, Table 3; Figure 4), elevation had a significant negative effect on KDE intensity, with a 100 m increase associated with a 2.6% decrease in KDE. Built-up areas showed a strong negative association, with a 10% increase corresponding to a reduction of approximately a 31% in KDE. In contrast, bare land cover was positively associated with KDE, increasing intensity by approximately 6% per 10% increase. The southern stratum exhibited nearly 48% higher KDE intensity compared with the northern reference area. Effect sizes were back-transformed and expressed as a percentage change in habitat use intensity, and the 95% confidence intervals are illustrated in Figure 4.

## 4. Discussion

The present study provides local density estimates and spatial habitat use patterns of *Capra aegagrus* within surveyed mountainous habitats of Batman Province using Line Transect Distance Sampling (LTDS) complemented by Kernel Density Estimation (KDE). By combining density estimation with spatial encounter analysis, this study offers an integrated evaluation of abundance and landscape-level habitat use within accessible mountain systems.

### 4.1. Distance Sampling Performance and Assumptions

LTDS provides a well-established framework for estimating densities of gregarious ungulates in open and semi-open habitats [49,50,51,52,53,54,55,56,57]. In rugged mountainous terrain, fully randomized transect placement is often constrained by accessibility and safety considerations [58,59,60,61]. Therefore, transects were implemented using a restricted systematic design within accessible habitats known to support wild goats. Consequently, the resulting estimates represent local ecological densities within surveyed mountainous areas rather than province-wide population parameters.

**Figure 5:**
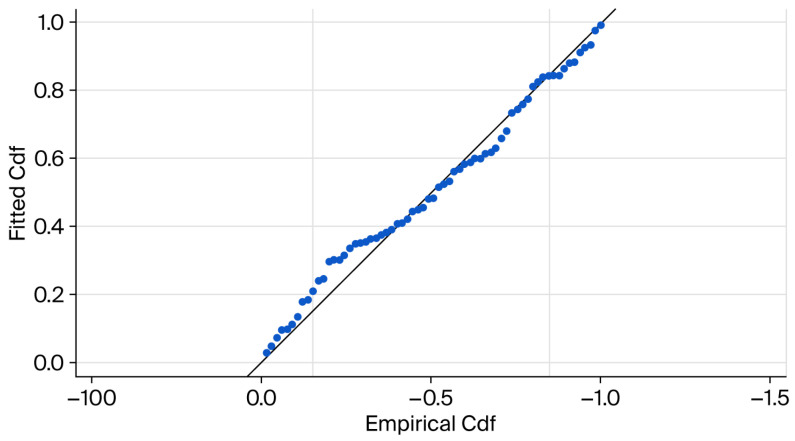
Empirical and fitted cumulative distribution functions for the cosine-adjusted half-normal detection function following left and right truncation. Points denote the empirical distribution, whereas the solid line denotes the fitted model.

The goodness-of-fit diagnostics (Kolmogorov–Smirnov, *p* = 0.35; Cramér–von Mises, *p* = 0.301) indicated that there was no significant deviation between the expecteded distance distributions and those that were observed (Figure 5). Detection probability declined smoothly with perpendicular distance, and post-truncation distance histograms were well represented by the fitted detection function. Although left truncation improved model fit and reduced potential bias from avoidance near the transect line, perfect detection at zero distance cannot be strictly assumed. Density estimates should therefore be interpreted cautiously as baseline indicators for monitoring.

Higher coefficients of variation in the northern stratum reflect a smaller effective sample size, highlighting the influence of encounter frequency on precision. Pooled analyses produced narrower confidence intervals, demonstrating improved stability when data were combined across strata.

### 4.2. Spatial Habitat Use Patterns

KDE visualized relative encounter intensity within surveyed mountainous habitats, identifying localized concentration areas in both northern and southern systems. Low kernel intensity values outside these areas reflect limited sampling coverage rather than confirmed ecological absence; therefore, they should not be interpreted as true absence.

The spatial modelling results indicate that habitat use is strongly structured across the landscape. Elevation showed a modest negative association with encounter intensity, suggesting selective use within an optimal altitudinal range. Slope exhibited a positive association, consistent with rugged terrain providing refuge from disturbance. The built land proportion demonstrated a pronounced negative effect, highlighting its sensitivity to anthropogenic influence. Although the southern stratum exhibited higher relative encounter intensity, density confidence intervals overlapped between strata, indicating comparable local densities.

### 4.3. Comparison with Previous Studies

Studies performed previously in Batman Province reported lower mean densities using direct observation and camera-trap-based approaches [35]. The LTDS-based estimate obtained here (6.47 individuals/km^2^) is higher, yet the total population size remains within previously reported confidence intervals. This variation likely reflects methodological differences, sampling intensity, and spatial coverage rather than true demographic change.

Across Türkiye, reported densities of *C. aegagrus* vary considerably among regions depending on habitat structure and survey design [36,37,38,39,40]. The present estimate falls within the upper range reported for mountainous and semi-protected systems [41]. Likewise, international comparisons demonstrate strong regional variability [42], underscoring the context-dependent nature of density estimates for this species.

### 4.4. Conservation Implications

South-Eastern Türkiye represents a biogeographically transitional and increasingly fragmented landscape for *C. aegagrus*, particularly following large-scale infrastructure development such as the Ilısu Dam. Quantitative density estimates from this region remain limited, and the present study provides baseline data for future monitoring.

While local densities were comparable between surveyed mountain systems, the Ilısu Dam reservoir now physically separates northern and southern habitats. Large reservoirs may function as landscape barriers, potentially limiting long-term connectivity. Although no density differentiation is currently evident, continued monitoring is necessary to evaluate potential demographic or genetic consequences of fragmentation.

From a management perspective, conservation efforts should prioritize maintaining mountainous forest–rocky mosaics that support consistent encounter intensity while minimizing anthropogenic disturbance [62,63,64]. Expanding spatial coverage and sampling effort would improve precision and enable broader inference. Future studies incorporating multivariate or hierarchical modelling frameworks may further clarify the combined effects of environmental gradients and human pressures on local encounter intensity.

## 5. Conclusions

The integration of Line Transect Distance Sampling and spatial regression analysis revealed consistent patterns were revealed that linked local density and spatial habitat use of *Capra aegagrus* within surveyed mountainous habitats of Batman Province. The estimated density (6.47 individuals/km^2^) falls within the range reported for comparable mountainous ecosystems in Türkiye; however, this should be interpreted as a local ecological baseline rather than a province-wide population estimate.

Habitat use intensity was spatially structured and primarily associated with topography and land-cover composition. Built-up areas were negatively associated with encounter intensity, underscoring sensitivity to human disturbance, whereas slope and bare land were positively associated with habitat use. Although density estimates were broadly comparable between northern and southern strata, continued monitoring is necessary to evaluate potential long-term effects of landscape fragmentation.

Given the constrained survey design and the ecological focus on accessible mountainous habitats, the results of this study provide a spatially explicit baseline suitable for future monitoring rather than definitive population-size estimates. The integrating of spatial modelling frameworks with standardized density estimation provides a practical approach for conservation planning in rugged, fragmented mountain systems.

## Figures and Tables

**Table 1:** Summary of the study area, sampling effort, and encounter statistics for Line Transect Distance Sampling (LTDS) surveys conducted in the north and south strata and across the total study area. (*n* represents the number of independent encounters with *Capra aegagrus*, and *k* denotes the number of transect walks conducted during the survey period.

	North	South	Total
Study area (km^2^)	190	241	431
Covered area (km^2^)	36.57	76.42	112.99
Sampling effort (km)	91.75	191.72	283.47
***n***	22	43	65
***k***	88	154	242

**Table 2:** Parameter estimates, standard errors (SEs), coefficients of variation (%CV), and 95% confidence intervals (CIs) derived from the Line Transect Distance Sampling (LTDS) analysis for *Capra aegagrus* in the north and south strata and for the total study area. Parameters include encounter rate of cluster [ER(S)], encounter rate of individuals (ER), density estimates of clusters (DS), density estimates of individuals (D), mean and expected cluster size [E(S), M(S)], and estimated population size (N).

	Parameter	Estimate	SE	%CV	95% CI
North	ER(S)	0.23	0.046	19.24	0.14–0.32
	ER	1.97	0.455	23.05	1.08–2.86
	DS	0.71	0.268	37.75	0.34–1.46
	D	5.85	2.329	39.82	2.74–12.50
	E(S)	8.22	1.104	13.42	6.06–10.38
	M(S)	8.23	1.124	13.65	6.03–10.43
	N*	1111	442.550	39.82	519–2375
South	ER(S)	0.22	0.030	13.27	0.16–0.28
	ER	2.08	0.368	17.74	1.36–2.80
	DS	0.75	0.191	25.47	0.45–1.23
	D	6.95	1.951	28.06	4.04–11.98
	E(S)	9.25	1.108	11.97	7.08–11.42
	M(S)	9.26	1.117	12.06	7.07–11.45
	N	1675	470.317	28.06	972–2887
Total	ER(S)	0.23	0.026	11.37	0.18–0.28
	ER	2.03	0.287	14.15	1.47–2.59
	DS	0.73	0.159	21.75	0.48–1.22
	D	6.47	1.498	23.17	4.11–10.16
	E(S)	8.81	0.780	9.07	7.28–10.34
	M(S)	8.91	0.828	9.29	7.29–10.53
	N	2787	645.793	23.17	1773–4379

N* denotes the estimated total abundance of individuals within the sampled strata, derived from the density estimates and the total area size.

**Table 3:** Parameter estimates from the final reduced Spatial Error Model explaining variation in KDE intensity. Elevation effects are scaled per 100 m increase; land-cover variables are scaled per 10% increase.

Variable	Estimate (β)	SE	z	*p*-Value	% Change (Scaled)	Lower CI	Upper CI
Elevation	−0.00026	0.00004	−6.97	<0.001	−2.6% (per 100 m)	−0.03349922	−0.01879273
Slope	0.0067	0.0030	2.21	0.027	+0.67% (per 1°)	0.07724063	1.27363057
Settlements (%)	−3.70	0.97	−3.81	<0.001	−31% (per 10%)	−99.63419282	−83.44699083
Bare land (%)	0.59	0.20	2.97	0.003	+6% (per 10%)	21.94465314	164.38371003
Southern stratum	0.39	0.12	3.36	<0.001	+48%	17.74893561	85.84931052

## Data Availability

All data generated or analysed during this study are included in this published article (and its Supplementary Information Files).

## Supplementary Materials

The following supporting information can be downloaded at: https://www.mdpi.com/article/10.3390/life16030432/s1, Table S1. The table below shows the coordinates, elevations, and lengths of the transects in the study area for the wild goat *Capra aegagrus* observations conducted using the line transect method, as well as the coordinates where the species was observed in each encounter, the distance and angle between them, and the observer’s coordinates and the distance and angle to the species. Additionally, the table includes information such as the date, time, number, and structure of individuals/groups (male, female, and young) for each encounter; Table S2. Spatial diagnostic statistics and model comparison for Ordinary Least Squares (OLS) and spatial regression models explaining variation in log-transformed KDE intensity (*n* = 87). Moran’s I values are calculated from model residuals under randomisation. Spatial parameters are λ for Spatial Error Models (SEM) and ρ for Spatial Autoregressive (SAR) and Spatial Durbin (SDM) models. The lowest AIC value indicates the best-supported model; Table S3. Descriptive statistics of KDE intensity and environmental predictors used in spatial regression models (*n* = 87). Land-cover variables represent proportional cover within sampling units.
