# Supplementary material for: Integrating Line Transect Distance Sampling and Spatial Analysis to Assess Local Density and Habitat Use of Capra aegagrus in Batman Province, Türkiye
Source: Life (Basel). 2026 Mar 6;16(3):432. doi: 10.3390/life16030432 (PMC13027745; doi:10.3390/life16030432)
Supplement: Supplementary file 1 [file life-16-00432-s001.zip › Table S3.pdf]

Table S3. Descriptive statistics of KDE intensity and environmental predictors used in spatial regression models (n = 87). Land-cover variables represent proportional cover within sampling units.

Variable	Min	1st Quartile	Median	Mean	3rd Quartile	Max
KDE intensity	0.0568	0.0849	0.1021	0.1007	0.1144	0.1425
Elevation (m)	500	816.5	1061	1042.7	1187.5	1799
Slope (°)	3.66	7.06	9.14	9.37	11.84	18.86
Water bodies (%)	0.000	0.000	0.000	0.033	0.000	0.404
Settlements (%)	0.000	0.00005	0.0046	0.0069	0.0150	0.0385
Forest cover (%)	0.000	0.0021	0.0374	0.0739	0.1021	0.3279
Agricultural land (%)	0.000	0.0009	0.0055	0.0179	0.0188	0.1937
Bare land (%)	0.00005	0.0023	0.0106	0.0446	0.0646	0.1795
