# Supplementary material for: Integrating Line Transect Distance Sampling and Spatial Analysis to Assess Local Density and Habitat Use of Capra aegagrus in Batman Province, Türkiye
Source: Life (Basel). 2026 Mar 6;16(3):432. doi: 10.3390/life16030432 (PMC13027745; doi:10.3390/life16030432)
Supplement: Supplementary file 1 [file life-16-00432-s001.zip › Table S1.pdf]

Table S1. The table below shows the coordinates, elevations, and lengths of the transects in the study area for the wild goat *Capra aegagrus* observations conducted using the Line transect method, as well as the coordinates where the species was observed in each encounter, the distance and angle between them, and the observer's coordinates and the distance and angle to the species. Additionally, the table includes information such as the date, time, number, and structure of individuals/groups (male, female, adult, young adult, and juvenile) for each encounter.

Sıra No	Region	Transects	Effort (km)	Observation No	Transect Line Coordinates (Degree)				Animals coordinates		Observers coordinates		Observers altitude	Animals altitude	Slope (%)	Radial Distance (*r*)	Angel	Perpendicular Distance (x)	Date	Time	Male	Female	Young	Cluster Size
					Start_longitude	Start_latitude	End_longitude	End_latitude	Longitude	Latitude	Longitude	Latitude												
1	North	**T1**	0.689	T1_01	38,3992	41,4660	38,3939	41,4695	38,3985	41,4691	38,3979	41,4667	923	1036	37,8	219,27	27,16	196,44	2022.09.07	09:17		3	9	12
2	North	**T1**	0.689	T1_02	38,3992	41,4660	38,3939	41,4695	38,3980	41,4695	38,3970	41,4680	925	1014	34,9	175,94	26,74	175,82	2023.05.21	18:21	1	3	9	13
3	North	**T1**	0.689	T1_03	38,3992	41,4660	38,3939	41,4695	38,3954	41,4702	38,3950	41,4692	922	989	20,1	97,60	34,13	40,3	2021.04.11	16:48		2	6	8
4	North	**T1**	0.689	T1_04	38,3992	41,4660	38,3939	41,4695	38,3967	41,4702	38,3958	41,4689	928	1009	29,3	157,68	27,09	146,01	2020.12.21	08:14	2	2	5	9
5	North	**T2**	0.700	T2_01	38,4254	41,4645	38,4219	41,4705	38,4248	41,4673	38,4241	41,4657	1132	1222	35,6	159,42	29,3	136,13	2021.06.14	13:46	2	2	5	9
6	North	**T2**	0.700	T2_02	38,4254	41,4645	38,4219	41,4705	38,4240	41,4692	38,4227	41,4675	1106	1212	35,6	201,76	27,61	124,81	2022.08.12	10:28	1	1	2	4
7	North	**T3**	0.803	T3_01	38,4196	41,4994	38,4195	41,5081	38,4216	41,5007	38,4193	41,4999	1226	1386	42,0	265,83	30,85	143,08	2021.03.09	07:53		1	3	4
8	North	**T3**	0.803	T3_02	38,4196	41,4994	38,4195	41,5081	38,4214	41,5041	38,4190	41,5036	1217	1373	41,1	268,27	30,01	264,67	2021.04.07	15:49	2	5	9	16
9	North	**T3**	0.803	T3_03	38,4196	41,4994	38,4195	41,5081	38,4209	41,5065	38,4194	41,5072	1221	1331	33,9	171,52	32,42	144,52	2022.09.15	18:37		1	2	3
10	North	**T3**	0.803	T3_04	38,4196	41,4994	38,4195	41,5081	38,4203	41,5016	38,4190	41,5016	1250	1303	29,1	137,60	21,04	112,13	2023.02.19	09:08		2	4	6
11	North	**T3**	0.803	T3_05	38,4196	41,4994	38,4195	41,5081	38,4209	41,5048	38,4192	41,5046	1223	1332	36,2	190,15	29,66	187,03	2021.11.08	08:42	4	5	6	15
12	North	**T4**	0.469	T4_01	38,4194	41,5183	38,417	41,5224	38,4197	41,5201	38,4187	41,5192	1351	1416	22,1	137,00	25,31	24,52	2022.06.04	06:53		1	2	3
13	North	**T4**	0.469	T4_02	38,4194	41,5183	38,417	41,5224	38,4196	41,5221	38,4177	41,5214	1355	1458	34,2	218,00	25,22	19,33	2021.03.17	16:24		2	4	6
14	North	**T4**	0.469	T4_03	38,4194	41,5183	38,417	41,5224	38,4197	41,5213	38,4183	41,5203	1353	1440	32,0	184,00	25,24	19,27	2022.05.13	19:07	2	5	11	18
15	North	**T4**	0.469	T4_04	38,4194	41,5183	38,417	41,5224	38,4191	41,5227	38,4173	41,5221	1369	1462	32,2	202,00	24,66	91,89	2022.08.27	18:35	1	2	2	5
16	North	**T5**	1.300	T5_01	38,3212	41,5584	38,3198	41,5706	38,3235	41,5650	38,3213	41,5659	1138	1249	34,5	244,00	24,4	162,46	2020.11.02	12:20		4	5	9
17	North	**T5**	1.300	T5_02	38,3212	41,5584	38,3198	41,5706	38,3221	41,5629	38,3206	41,5652	1120	1249	38,5	253,00	26,92	247,06	2021.04.10	10:34	1	3	2	6
18	North	**T5**	1.300	T5_03	38,3212	41,5584	38,3198	41,5706	38,3252	41,5677	38,3222	41,5678	1183	1301	30,1	328,00	19,77	260,45	2021.01.28	16:19		2		2
19	North	**T6**	2.440	T6_01	38,3486	41,5654	38,3299	41,5748	38,3392	41,5723	38,3371	41,5700	1285	1425	35,2	313,00	24,04	277,19	2022.03.30	14:35			2	2
20	North	**T6**	2.440	T6_02	38,3486	41,5654	38,3299	41,5748	38,3437	41,5685	38,3424	41,5659	1401	1582	34,0	269,00	33,62	216,11	2021.05.06	09:48	4	8	8	20
21	North	**T6**	2.440	T6_03	38,3486	41,5654	38,3299	41,5748	38,3446	41,5639	38,3446	41,5649	1460	1514	19,5	84,50	32,33	66,89	2022.10.17	08:51		3		3
22	North	**T6**	2.440	T6_04	38,3486	41,5654	38,3299	41,5748	38,3430	41,5705	38,3415	41,5663	1356	1643	42,5	408,24	34,74	72,99	2022.08.28	19:20	2	3	7	12

Sıra No	Region	Transects	Effort (km)	Observation No	Transect Line Coordinates (Degree)				Animals coordinates		Observers coordinates		Observers altitude	Animals altitude	Slope (%)	Radial Distance (*r*)	Angel	Perpendicular Distance (x)	Date	Time	Male	Female	Young	Cluster Size
					Start_longitude	Start_latitude	End_longitude	End_latitude	Longitude	Latitude	Longitude	Latitude												
23	North	**T7**	1.007	T7_01	38,2589	41,6002	38,2544	41,597	38,2544	41,6011	38,2542	41,5987	1038	1114	29,3	214,37	19,5	130,25	2020.11.24	12:45		1		1
24	North	**T7**	1.007	T7_02	38,2589	41,6002	38,2544	41,597	38,2573	41,6020	38,2584	41,5991	1109	1225	32,2	283,21	22,24	69,18	2021.01.28	14:39		2	5	7
25	North	**T7**	1.007	T7_03	38,2589	41,6002	38,2544	41,597	38,2570	41,6009	38,2567	41,5986	1067	1162	35,1	204,42	24,86	54,59	2021.04.10	17:25	1	2	2	5
26	North	**T7**	1.007	T7_04	38,2589	41,6002	38,2544	41,597	38,2557	41,6023	38,2554	41,5994	1061	1209	41,2	263,68	29,16	204,11	2022.06.11	07:26	3	4	12	19
27	North	**T8**	0.933	T8_01	38,2614	41,6133	38,2607	41,6064	38,2568	41,6092	38,2593	41,6112	1185	1311	33,0	318,29	21,57	131,15	2021.02.14	13:50	1			1
28	North	**T8**	0.933	T8_02	38,2614	41,6133	38,2607	41,6064	38,2620	41,6052	38,2608	41,6066	1075	1177	44,7	173,00	30,34	151,91	2021.07.05	11:55		2	8	10
29	North	**T8**	0.933	T8_03	38,2614	41,6133	38,2607	41,6064	38,2597	41,6145	38,2604	41,6108	1158	1283	32,2	327,46	20,87	295,27	2021.01.28	12:40		3	4	7
30	North	**T8**	0.933	T8_04	38,2614	41,6133	38,2607	41,6064	38,2590	41,6138	38,2595	41,6113	1180	1277	34,5	223,84	23,38	220,27	2021.05.06	07:52	1	2	5	8
31	South	**T9**	0.634	T9_01	37,6879	41,6091	37,6842	41,603	37,6828	41,6049	37,6850	41,6045	895	1023	44,3	234,27	28,52	57,4	2020.10.13	13:20		2	2	4
32	South	**T9**	0.634	T9_02	37,6879	41,6091	37,6842	41,603	37,6837	41,6073	37,6855	41,6056	889	1007	35,5	246,00	25,55	100,36	2023.12.16	09:41		2	1	3
33	South	**T9**	0.634	T9_03	37,6879	41,6091	37,6842	41,603	37,6820	41,6035	37,6847	41,6039	903	1057	40,1	277,61	28,88	158,17	2022.08.27	19:05	1			1
34	South	**T9**	0.634	T9_04	37,6879	41,6091	37,6842	41,603	37,6857	41,6086	37,6867	41,6071	898	975	30,1	160,74	25,52	61,23	2021.10.15	09:26	2	5	11	18
35	South	**T10**	1.038	T10_01	37,7063	41,6035	37,7002	41,6109	37,7007	41,6056	37,7025	41,6072	699	776	29,7	239,41	17,82	205,49	2021.03.15	10:50		4	5	9
36	South	**T10**	1.038	T10_02	37,7063	41,6035	37,7002	41,6109	37,7036	41,6096	37,7021	41,6085	707	828	38,2	191,00	32,11	122,49	2022.04.16	15:43	1	2	6	9
37	South	**T10**	1.038	T10_03	37,7063	41,6035	37,7002	41,6109	37,7022	41,6123	37,7014	41,6098	710	826	34,5	230,12	26,66	229,91	2022.07.02	18:50		2	6	8
38	South	**T11**	1.266	T11_01	37,7084	41,5809	37,6983	41,5766	37,6987	41,5801	37,7011	41,5792	749	838	27,6	267,37	18,4	116,76	2021.11.10	14:30		1		1
39	South	**T11**	1.266	T11_02	37,7084	41,5809	37,6983	41,5766	37,7024	41,5824	37,7049	41,5810	715	785	21,3	282,13	13,93	276,16	2021.06.10	07:46		2	2	4
40	South	**T11**	1.266	T11_03	37,7084	41,5809	37,6983	41,5766	37,6997	41,5812	37,7015	41,5796	744	845	33,8	216,54	24,94	41,21	2023.12.16	10:37			2	2
41	South	**T11**	1.266	T11_04	37,7084	41,5809	37,6983	41,5766	37,6970	41,5794	37,6989	41,5771	788	909	32,6	279,28	23,38	274,62	2022.05.21	15:26	3	4	5	12
42	South	**T11**	1.266	T11_05	37,7084	41,5809	37,6983	41,5766	37,7007	41,5822	37,7027	41,5802	732	856	35,2	268,11	24,76	98,02	2022.08.27	17:25		1	2	3
43	South	**T12**	1.187	T12_01	37,7037	41,4671	37,6952	41,4675	37,6994	41,4677	37,6997	41,4704	602	664	25,5	237,05	14,65	206,21	2020.12.20	12:25	1	4	6	11
44	South	**T12**	1.187	T12_02	37,7037	41,4671	37,6952	41,4675	37,7009	41,4718	37,7021	41,4692	582	663	25,6	286,25	15,79	24,5	2021.02.14	13:40	1	3	6	10
45	South	**T12**	1.187	T12_03	37,7037	41,4671	37,6952	41,4675	37,6978	41,4709	37,6977	41,4683	597	640	20,8	218,34	11,14	216,06	2022.11.04	09:24	1	4	5	10
46	South	**T13**	0.860	T13_01	37,7	41,4594	37,6924	41,4627	37,6977	41,4568	37,6982	41,4593	621	722	35,2	219,29	24,67	98,1	2021.09.12	11:10	1	8	9	18
47	South	**T13**	0.860	T13_02	37,7	41,4594	37,6924	41,4627	37,6968	41,4633	37,6966	41,4603	611	666	29,9	266,02	11,68	206,1	2023.01.07	08:55		1	1	2

Sıra No	Region	Transects	Effort (km)	Observation No	Transect Line Coordinates (Degree)				Animals coordinates		Observers coordinates		Observers altitude	Animals altitude	Slope (%)	Radial Distance (r)	Angel	Perpendicular Distance (x)	Date	Time	Male	Female	Young	Cluster Size
					Start_longitude	Start_latitude	End_longitude	End_latitude	Longitude	Latitude	Longitude	Latitude												
48	South	**T13**	0.860	T13_03	37,7	41,4594	37,6924	41,4627	37,6986	41,4618	37,6973	41,4596	609	707	27,6	240,18	22,16	40,79	2022.03.22	10:30		4	5	9
49	South	**T14**	1.484	T14_01	37,6976	41,4199	37,686	41,4232	37,6926	41,4179	37,6930	41,4205	760	822	32,2	217,42	15,91	43,56	2022.03.08	13:30	2	3	3	8
50	South	**T14**	1.484	T14_02	37,6976	41,4199	37,686	41,4232	37,6898	41,4189	37,6894	41,4206	811	827	21,9	156,00	5,86	64,64	2022.10.07	10:19	5	4	9	18
51	South	**T14**	1.484	T14_03	37,6976	41,4199	37,686	41,4232	37,6877	41,4248	37,6867	41,4227	860	914	22,6	210,54	14,38	204,29	2021.08.12	08:45	1	5	6	12
52	South	**T14**	1.484	T14_04	37,6976	41,4199	37,686	41,4232	37,6900	41,4235	37,6883	41,4214	860	976	19,3	257,04	24,23	201,17	2022.06.10	12:30		9	13	22
53	South	**T15**	1.189	T15_01	37,6626	41,3832	37,6593	41,3734	37,6647	41,3766	37,6637	41,3782	1100	1145	22,6	172,34	14,63	151,86	2020.11.20	10:30	1	10	8	19
54	South	**T15**	1.189	T15_02	37,6626	41,3832	37,6593	41,3734	37,6607	41,3784	37,6622	41,3773	1150	1208	26,5	184,52	17,44	182,15	2021.01.15	13:24	2			2
55	South	**T15**	1.189	T15_03	37,6626	41,3832	37,6593	41,3734	37,6588	41,3757	37,6606	41,3762	1176	1239	21,6	211,27	16,6	163,96	2023.03.15	15:46		1	3	4
56	South	**T15**	1.189	T15_04	37,6626	41,3832	37,6593	41,3734	37,6609	41,3740	37,6605	41,3752	1187	1214	8,7	109,08	13,9	106,01	2023.05.19	18:10		1	1	2
57	South	**T15**	1.189	T15_05	37,6626	41,3832	37,6593	41,3734	37,6598	41,3776	37,6611	41,3764	1168	1219	19,8	161,28	17,54	155,92	2021.10.21	09:36	2	3	7	12
58	South	**T15**	1.189	T15_06	37,6626	41,3832	37,6593	41,3734	37,6622	41,3754	37,6617	41,3772	1158	1202	19,0	155,37	15,81	15,14	2022.08.06	17:48	1	2	7	10
59	South	**T16**	1.314	T16_01	37,6639	41,3969	37,658	41,3882	37,6614	41,3957	37,6627	41,3935	1065	1133	25,3	234,12	16,19	108,29	2022.08.06	11:25	1	10	12	23
60	South	**T16**	1.314	T16_02	37,6639	41,3969	37,658	41,3882	37,6574	41,3895	37,6596	41,3882	1064	1123	20,6	263,00	12,64	19,89	2022.12.20	10:30	2	12	16	30
61	South	**T16**	1.314	T16_03	37,6639	41,3969	37,658	41,3882	37,6606	41,3916	37,6629	41,3902	1042	1106	21,3	273,81	13,15	151,7	2023.05.19	07:55		2	5	7
62	South	**T16**	1.314	T16_04	37,6639	41,3969	37,658	41,3882	37,6598	41,3903	37,6618	41,3889	1039	1093	20,7	238,14	12,77	49,1	2021.10.21	12:21		2	5	7
63	South	**T16**	1.314	T16_05	37,6639	41,3969	37,658	41,3882	37,6586	41,3898	37,6603	41,3882	1051	1104	20,5	231,19	12,91	77,81	2022.02.10	07:16	1	1	2	4
64	South	**T17**	1.165	T17_01	37,6225	41,3191	37,6163	41,3265	37,6190	41,3283	37,6183	41,3262	1009	1138	36,0	204,37	32,02	116,36	2021.12.07	09:45		1	3	4
65	South	**T17**	1.165	T17_02	37,6225	41,3191	37,6163	41,3265	37,6209	41,3268	37,6201	41,3244	986	1069	27,1	233,64	19,54	148,63	2024.03.09	12:45		1		1
66	South	**T17**	1.165	T17_03	37,6225	41,3191	37,6163	41,3265	37,6185	41,3290	37,6177	41,3268	1022	1118	34,7	210,39	24,47	129,66	2021.09.09	17:12	4	2	9	15
67	South	**T18**	0.704	T18_01	37,6131	41,3379	37,6118	41,3453	37,6141	41,3414	37,6127	41,3415	1172	1240	27,7	150,26	24,29	111,87	2020.11.08	15:05			3	3
68	South	**T18**	0.704	T18_02	37,6131	41,3379	37,6118	41,3453	37,6139	41,3396	37,6128	41,3393	1154	1239	24,3	116,32	35,73	106,99	2023.01.13	12:40		3	1	4
69	South	**T18**	0.704	T18_03	37,6131	41,3379	37,6118	41,3453	37,6132	41,3443	37,6117	41,3450	1164	1231	26,2	163,08	22,3	49,38	2022.07.05	18:25		1	6	7
70	South	**T18**	0.704	T18_04	37,6131	41,3379	37,6118	41,3453	37,6138	41,3420	37,6122	41,3426	1167	1231	28,6	182,37	19,32	82,73	2022.11.11	08:42		1	4	5
71	South	**T19**	0.793	T19_01	37,6139	41,357	37,6078	41,3594	37,6121	41,3592	37,6109	41,3569	1062	1112	21,0	247,15	11,44	223,6	2022.06.02	10:30		3	3	6
72	South	**T19**	0.793	T19_02	37,6139	41,357	37,6078	41,3594	37,6113	41,3593	37,6102	41,3573	1046	1088	17,9	202,21	11,73	149,76	2022.02.17	15:35	2			2

Sıra No	Region	Transects	Effort (km)	Observation No	Transect Line Coordinates (Degree)				Animals coordinates		Observers coordinates		Observers altitude	Animals altitude	Slope (%)	Radial Distance (r)	Angel	Perpendicular Distance (x)	Date	Time	Male	Female	Young	Cluster Size
					Start_longitude	Start_latitude	End_longitude	End_latitude	Longitude	Latitude	Longitude	Latitude												
73	South	**T19**	0.793	T19_03	37,6139	41,357	37,6078	41,3594	37,6109	41,3602	37,6093	41,3582	1039	1088	18,3	247,27	11,21	241,73	2023.05.20	16:27	1	2	3	6
74	South	**T19**	0.793	T19_04	37,6139	41,357	37,6078	41,3594	37,6107	41,3605	37,6083	41,3592	1025	1081	19,4	287,05	11,04	286,79	2022.11.11	07:10	2	4	11	17
75	South	**T20**	0.743	T20_01	37,667	41,5024	37,6626	41,5036	37,6626	41,5058	37,6643	41,5038	1189	1263	27,7	256,31	16,1	97,27	2022.06.08	13:05	1	8	19	28
76	South	**T20**	0.743	T20_02	37,667	41,5024	37,6626	41,5036	37,6644	41,5065	37,6654	41,5035	1157	1249	24,5	284,24	17,92	227,2	2022.10.14	12:28	2	5	15	22
77	South	**T21**	2.479	T21_01	37,7374	41,4594	37,7376	41,4864	37,7391	41,4612	37,7376	41,4609	549	597	21,8	162,45	16,45	110,19	2020.11.10	10:45		5	3	8
78	South	**T21**	2.479	T21_02	37,7374	41,4594	37,7376	41,4864	37,7396	41,4802	37,7376	41,4801	548	613	21,8	203,47	17,71	185,24	2021.09.18	13.30		12	12	24
79	South	**T21**	2.479	T21_03	37,7374	41,4594	37,7376	41,4864	37,7395	41,4665	37,7372	41,4660	530	609	26,5	253,00	17,33	252,65	2022.04.24	12:25	1	15	16	32
80	South	**T21**	2.479	T21_04	37,7374	41,4594	37,7376	41,4864	37,7402	41,4701	37,7381	41,4691	562	653	31,9	230,38	21,52	103,78	2022.01.14	09:41		3	6	9
81	South	**T21**	2.479	T21_05	37,7374	41,4594	37,7376	41,4864	37,7397	41,4722	37,7374	41,4718	539	621	28,0	234,76	19,24	88,69	2022.08.29	19:25	1	4	9	14
82	South	**T21**	2.479	T21_06	37,7374	41,4594	37,7376	41,4864	37,7391	41,4758	37,7376	41,4762	553	622	27,2	166,39	22,49	78,95	2021.10.15	08:27	1	2	3	6
83	South	**T22**	2.573	T22_01	37,7319	41,5401	37,7332	41,5562	37,7339	41,5375	37,7316	41,5380	515	580	22,4	255,37	14,28	252,89	2020.12.07	16:00		4	9	13
84	South	**T22**	2.573	T22_02	37,7319	41,5401	37,7332	41,5562	37,7325	41,5441	37,7317	41,5450	517	546	0,6	112,87	14,41	108,83	2023.02.24	09:15		1	3	4
85	South	**T22**	2.573	T22_03	37,7319	41,5401	37,7332	41,5562	37,7343	41,5514	37,7329	41,5512	515	568	19,3	153,21	19,07	32,88	2022.03.08	14:27		1	3	4
86	South	**T22**	2.573	T22_04	37,7319	41,5401	37,7332	41,5562	37,7342	41,5482	37,7326	41,5489	526	567	19,2	169,33	13,61	146,18	2022.05.17	08:43	1	1	3	5
87	South	**T22**	2.573	T22_05	37,7319	41,5401	37,7332	41,5562	37,7354	41,5536	37,7337	41,5542	535	629	32,7	194,17	25,76	113,37	2022.07.01	18:25		3	7	10
																					**73**	**272**	**461**	**806**
