# Supplementary material for: Integrating Line Transect Distance Sampling and Spatial Analysis to Assess Local Density and Habitat Use of Capra aegagrus in Batman Province, Türkiye
Source: Life (Basel). 2026 Mar 6;16(3):432. doi: 10.3390/life16030432 (PMC13027745; doi:10.3390/life16030432)
Supplement: Supplementary file 1 [file life-16-00432-s001.zip › Table S2.pdf]

Table S2. Spatial diagnostic statistics and model comparison for Ordinary Least Squares (OLS) and spatial regression models explaining variation in log-transformed KDE intensity (n = 87). Moran's I values are calculated from model residuals under randomisation. Spatial parameters are λ for Spatial Error Models (SEM) and ϱ for Spatial Autoregressive (SAR) and Spatial Durbin (SDM) models. The lowest AIC value indicates the best-supported model.

Model	Moran's I	z-value	p-value	Spatial parameter	Spatial parameter p-value	AIC
OLS	0.718	10.39	<0.001	—	—	-92.63
SAR	0.387	6.36	<0.001	$\varrho = 0.86$*	<0.001	-220.95
SDM	0.166	2.65	0.004	$\varrho = 0.88$*	<0.001	-247.14
SEM	0.231	3.63	<0.001	$\lambda = 0.92$	<0.001	-249.42
Reduced SEM	0.232	3.64	<0.001	$\lambda = 0.92$	<0.001	**-254.59**
